# Potential of *Eucalyptus camaldulensis* for phytostabilization and biomonitoring of trace-element contaminated soils

**DOI:** 10.1371/journal.pone.0180240

**Published:** 2017-06-30

**Authors:** Paula Madejón, Teodoro Marañón, Carmen M. Navarro-Fernández, María T. Domínguez, José M. Alegre, Brett Robinson, José M. Murillo

**Affiliations:** 1 Instituto de Recursos Naturales y Agrobiología de Sevilla, IRNAS, Seville, Spain; 2 Department of Soil and Physical Sciences, Lincoln University, Lincoln, Canterbury, New Zealand; RMIT University, AUSTRALIA

## Abstract

Soil pollution by trace elements (TEs) from mining and industrial activity is widespread and presents a risk to humans and ecosystems. The use of trees to immobilize TEs (phytostabilization) is a low-cost and effective method of soil remediation. We aimed to determine the chemical composition of leaves and flower buds of *Eucalyptus camaldulensis* in seven sites along the Guadiamar River valley (SW Spain), an area contaminated by a mine-spill in 1998. *E*. *camaldulensis* trees in the spill-affected area and adjacent non affected areas were growing on a variety of soils with pH from 5.6 to 8.1 with low concentration of plant nutrients. The spill affected soils contained up to 1069 mg kg^-1^ of As and 4086 mg kg^-1^ of Pb. *E*. *camaldulensis* tolerated elevated TE concentrations in soil and, compared to other species growing in the same environment, had low TE concentrations in the aerial portions. Besides tolerance to soil contamination, *E*. *camaldulensis* had low bioaccumulation coefficients for soil contaminants. TE concentrations in the aboveground portions were below levels reported to be toxic to plants or ecosystems. Flower buds had even lower TE concentrations than leaves. Despite the relatively low concentration of TEs in leaves they were significantly correlated with the soil extractable (0.01 M CaCl_2_) Cd, Mn and Zn (but not Cu and Pb). The general features of this tree species: tolerance to impoverished and contaminated soils, fast growth and deep root system, and low transfer of TEs from soil to aboveground organs makes it suitable for phytostabilization of soils contaminated by TEs. In addition, eucalyptus leaves could be used for biomonitoring the soil extractability of Cd, Mn and Zn but not Cu or Pb.

## Introduction

Soil pollution is a global environmental issue, with remediation costs in the EU alone estimated at €6 billion / yr [1]. This expense is justified because decontamination of heavy metals and other pollutants is imperative for eliminating or mitigating their negative effects on the environment and human health. Most of the physical and chemical methods for soil decontamination are prohibitively expensive for vast areas and may result in secondary pollution [2]. For these reasons, phytostabilization (use of plants to reduce the mobility and toxicity of soil contaminants) has been the subject of much research in the last two decades due to its low environmental disturbance and is relatively low cost compared to other remediation technologies [3, 4].

Phytostabilization may be especially useful for riparian areas, where the limited accessibility to riverbanks makes the application of other remediation approaches particularly difficult. Remediation of these areas should be prioritized, as they are highly dynamic systems where pollutants can be easily mobilized and transported by runoff waters following natural hydrological cycles [5], which can lead to the large-scale spread of pollutants across river basins. The use of woody plant species in these habitats is recommended because of their large root systems, which contribute to soil physical stabilization of riverbanks [6, 7].

The choice of plant species is one of the most important factors determining the success of phytostabilization [3]. Ideally, the species used should be native plants adapted to local environmental conditions [8], and they should exclude the entry of contaminants into the aboveground biomass.

The chemical composition of leaves or other plant parts can indicate the presence of contaminants in the environment and they are widely used as biomonitors [9–13]. An organism is considered as biomonitor when contains information on the quantitative aspects of the quality of the environment [10].

Due to a long history of mining activity, heavy metal pollution is a common environmental problem in Mediterranean countries [14]. Many recent studies have evaluated the feasibility of different native Mediterranean species for phytostabilization [15–17]. The restoration program established at the Guadiamar Valley (SW Spain) is one of the largest soil remediation initiatives conducted in the Mediterranean region, and phytostabilization was the most important technology [18]. Due to a mine spill, about 6 million m^3^ of sludge and acidic waters containing high concentrations As, Cd, Cu, Tl, and Zn, escaped into the Guadiamar River and contaminated ca. 2000 ha of arable and pastureland. Afterwards, the affected land was cleaned up, retired from cultivation, and planted with native shrubs and trees (see Materials and Method section for further details). Eucalyptus plantations (mainly of *Eucalyptus camaldulensis* and some *E*. *globulus*) were also removed, with exception of those trees growing in riparian areas with difficult access.

The wide distribution of *E*. *camaldulensis* trees within the Guadiamar valley, in a variety of soil types and with different levels of contamination, made that species suitable for studying plant-soil relationships. In general, eucalyptus species are good candidates for phytostabilization due to their rapid development of extensive root systems, which contributes to the physical stabilization of soils and riverbanks, their relatively high tolerance of TE contamination and nutrient imbalances in the growing substrate, and their ease of establishment on contaminated soils [19, 20]. In addition, their high essential oil content and lack of adapted herbivores outside their native range (Australia) may be an advantage for phytostabilization because there is less risk that the contaminants are transferred to the food chain. The possibility of gaining profit from the trees through the production of fuel, fibre, paper, timber or essential oils would allow a phytomanagement of the contaminated land [18] (see more information about the study species on Methods section). This field study fill a knowledge gap about the trace element uptake of eucalyptus trees growing on trace-elements contaminated soils, in a Mediterranean climate.

We aimed to elucidate the response of *Eucalyptus camaldulensis* to different levels of soil contamination and environmental conditions, in order to evaluate its potential for phytostabilization and as biomonitor of TEs. For comparative purposes we also studied a native willow (*Salix purpurea*) growing in the same contaminated sites.

Specifically, we sought to determine:

1. the range of environmental conditions where *E*. *camaldulensis* can be found in the Guadiamar valley; for example, whether the concentration of TEs in soil was above intervention values.
2. the effect of site conditions on the chemical composition of various tissues.
3. whether the chemical composition of *E*. *camaldulensis* leaf tissue indicates nutrient deficiencies or TE toxicity.
4. the bioaccumulation coefficients for soil-borne contaminants and suitability of *E*. *camaldulensis* for phytostabilization.
5. the suitability of *E*. *camaldulensis* for biomonitoring TEs in soil.

## Materials and methods

### Study area

The Guadiamar River valley (SW Spain) lies inside the Iberian Pyrite Belt, the largest massive sulphide province in Western Europe. The area has a Mediterranean climate with mild rainy winters and warm dry summers (an annual average of about 2900 h of sunshine and maximum values of solar radiation exceeding 1000 W m^-2^). Average annual temperature is 19°C (minimum monthly mean of 9°C in January, and maximum of 27°C in July) and annual average rainfall is 484 mm. Soils are mostly neutral or slightly alkaline in the southern part while they are acidic in the northern part. In 1998, the failure of a large mine tailing dam at Aznalcóllar (Seville) released about 6 million m^3^ of TE-polluted sludge into the Agrio and Guadiamar Rivers (see general description of the accident in [21]). After the accident, a reclamation project included the removal of the polluted topsoil, the application of soil amendments to promote TE stabilization, and the afforestation with native woody plant species [18]. Finally, the Regional Administration designed the remediated area (2706.8 ha) as protected, establishing the Guadiamar Green Corridor (GGC) [22].

Most of the remediation operations focused on the floodplain of both rivers, accompanied by a vast research and monitoring programme. Soil contamination was significantly reduced and the TE concentrations in planted shrubs and trees were mostly below guideline values [18]. The riverbanks and channels were more difficult than the floodplains to access and remediate. There were still some extremely acid soils (pH ≈ 3), due to the periodical oxidation of sludge that could not be removed, as well as considerable residual contamination [7].

Riparian forests in the Guadiamar River are mainly composed by eucalyptus (*Eucalyptus camaldulensis*) from old plantations, mixed with native white poplars (*Populus alba*), narrow-leafed ashes (*Fraxinus angustifolia*), field elms (*Ulmus minor*) and willows (*Salix* spp.).

### Study species

*Eucalyptus camaldulensis* Dehnh. (river red gum) is a tree native from Australia which has been widely planted around the world. It belongs to a genus, in the Myrtaceae family, with about 800 species, all of them restricted to Australasia. Eucalypts are evergreen, fast-growing, deep-rooting trees, tolerant of poor soil conditions, drought and moderate salinity [23]. Their plantations provide pulp, paper, timber, fuel wood, essential oil, medicine, bioenergy and nectar for honeybees, and mitigate human pressures on native forests. They are the most widely planted hardwood forest trees in the world, covering more than 15 million ha in 100 countries [24–26].

Their low tolerance to cold temperatures has restricted *E*. *camaldulensis* plantations in temperate environments. For example, in Europe they have been planted mainly in southern countries, like Portugal, Spain, Italy and Greece. About 633,000 ha planted in Spain, produce annually more than 4 million m^3^ of timber [27].

Eucalyptus species have been proposed for phytostabilization of contaminated soils because of their moderate to low rates of heavy metal accumulation into aboveground biomass, thus reducing the risk of transmission of contaminants into the food web [20, 28]. However, some eucalyptus trees could accumulate, under experimental conditions, a certain amount of heavy metals into their shoots [20, 29, 30]. The suitability of the chosen species should be evaluated for the particular conditions and the type of contaminants.

Outside of their native habitats, eucalyptus leaves, rich in secondary compounds, are rarely consumed by herbivores, so minimizing the potential risk for the food web. On the other hand, during flowering the nectar attracts bees and it is an important source of the commonly consumed and sold “eucalyptus honey” [31]. That food should be also monitored for heavy metals toxicity.

The massive plantations of exotic eucalypt trees out of their natural range have raised several environmental issues related to the destruction of native ecosystems and biodiversity loss, reduction of water availability (sometimes planted for draining swampy places), increasing fire risk, and promoting unsustainable timber production [32]. Nevertheless, in riparian areas the eucalyptus trees can contribute to soil phytostabilization, along with other species.

*Salix purpurea* L. (purple willow) is a deciduous arborescent shrub, up to 6 m height, growing on wet places, river and lake margins, distributed for almost all Europe, northern Africa and West Asia. Willows are fast-growing woody plants with the known capacity to accumulate significant amounts of certain TEs into aboveground biomass, and therefore they have been widely used for phytoremediation [33–37].

### Plant and soil sampling

**Fig 1:**
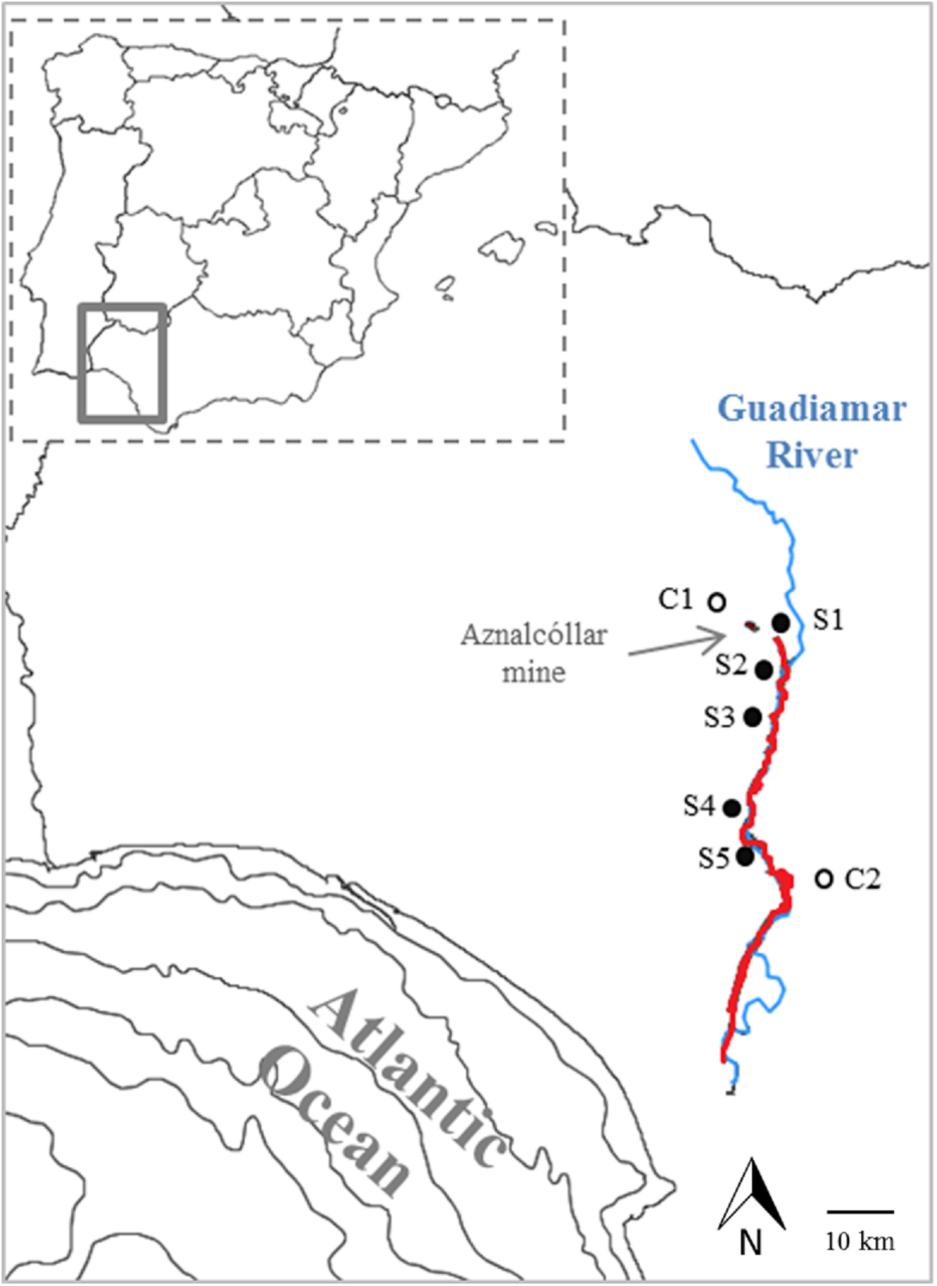
Map. Location of the seven sampling sites within the Guadiamar Basin, indicating the area affected by the mine spill in red.

**Fig 2:**
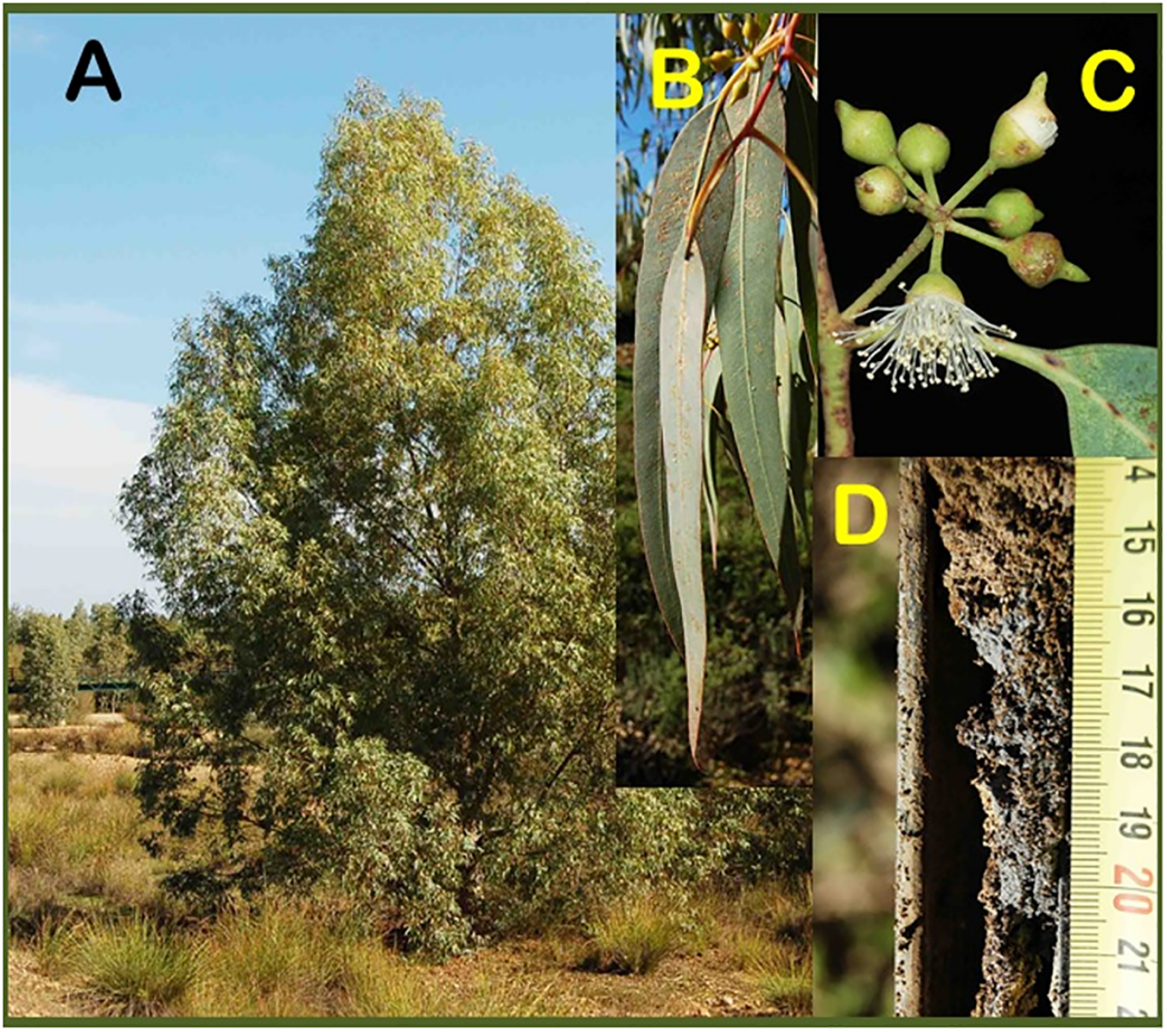
*Eucalyptus camaldulensis* at the Guadiamar River. A, sampled tree at site S2; B, details of leaves; C, details of flowers; D, soil core with sludge contamination from site S3.

Seven sampling sites were selected along the Guadiamar basin (Fig 1). Two of these sites were chosen in locations that were not affected by the mine-spill (C1 and C2, control sites). Site C1 was located in rocky slopes near an ephemeral stream, upwards of the Aznalcóllar mine. While site C2 was at the southern edge of the basin (29 km away from the mine), on a sandy plain outside of the affected area. Five sites (S1 to S5) were selected within the area affected by the spill. Site S1 was close to the broken dam, origin of the spill, in the river Agrio. Site S2 was located downwards (3 km away from the dam), where Agrio joins to the main Guadiamar river; that spot was highly contaminated during the cleaning operations, used as temporary storage of sludge (Fig 2A). Site S3 was located in a fenced control plot where the sludge was not removed, for research and monitoring purposes (13 km away from the dam). Sites S4 and S5 were located downstream, at 22 and 25 km respectively from the tailing pond (see coordinates in S1 Table).

At each site, three *E*. *camaldulensis* individuals were selected, more than 10 m apart, and marked. Sampling of leaves and soils occurred in November 2015 (17 years after the accident) and samples of flower buds were taken in April 2016 (Fig 2B and 2C). A general description of the sampled trees (height, trunk size (DBH), and leaf traits) is shown in the S2 Table. For comparison, *S*. *purpurea* trees were sampled at the same dates in the two sites (S1 and S2) where they coexisted with *E*. *camaldulensis*.

Leaves and flower buds were collected from the outer canopy at ca. 5m height. In general, leaves of the outer canopy tend to accumulate more mineral elements than those of the inner canopy, due to their higher transpiration rates [38]. The rhizosphere of each tree was sampled using a spiral auger at two depths (0–20 and 20–40 cm), at three points around the trunk base; they were combined to make one composite soil sample per tree and depth.

### Sample processing and analysis

Samples of leaves and flower buds were washed with distilled water for approximately 10 s, dried at 70°C for at least 48 h, and ground. Total N was measured by Kjeldahl digestion. TEs (As, Cd, Cu, Mn, Pb and Zn) and macronutrients (S, P, K, Ca and Mg) were determined by inductively coupled plasma spectrophotometry (ICP-OES: Varian ICP 720-ES: simultaneous ICP-OES with axially viewed plasma) after digestion of plant tissues by wet oxidation with concentrated HNO_3._ Plant digestion was carried out with a Digiprep MS block digestor (SPS Science) equipped with a temperature-time programmable controller and polypropylene digestion tubes. The accuracy of the analytical method was determined using a plant reference material: INCT-ONTL-5 (Tobacco leaves). Recovery rates for reference plant samples ranged between 90 and 103%.

A subsample of leaves from the same trees was used to measure two functional traits: leaf mass per area (LMA) as indicator of the tree growth rate [39] and the isotopic ratio of carbon (δ ^13^C) which provides a time-integrated measure of the tree water use efficiency [40]. A sample of 6–8 leaves by tree was scanned and analyzed with Image-Pro 4.5 (Media Cybernetic Inc. USA); they were oven-dried at 70°C, weighed and the ratio between mass and area was calculated; see methods in [41]. The isostopic ratio of carbon (δ ^13^C) was measured in combusted leaf samples at 1020°C using a continuous flow isotope-ratio mass spectrometry system by means of Flash HT Plus elemental analyzer coupled to a Delta-V Advantage isotope ratio mass spectrometer via a CONFLO IV interface (Thermo Fisher Scientific, Bremen, Germany). The analytical measurement error was ±0.1‰ for δ^13^C.

Soil samples were oven-dried at 40°C and sieved to <2 mm for physico-chemical analysis; a subsample was then ground to <60 μm for S and TE determination. Soil pH was measured in a 1:2.5 soil-water suspension by using a pH meter (CRISON micropH 2002). Size particle distribution was analyzed by the hydrometer method [42]. Total C content was determined by using a LECO TruSpec CHN analyzer, and carbonate content by the manometric method [43]. Organic C was calculated as the difference between total C and the C contained in carbonates. Total N was determined by Kjeldahl digestion [44]. Available P was estimated by the Olsen method [45], and available K was determined by atomic absorption spectrophotometry after extraction with 1M ammonium acetate [46].

Sulphur and total content of TEs in soils was determined by ICP-OES after digesting the samples (<60 μm fraction) with a mixture of concentrated HNO_3_ and HCl (‘aqua regia’) in a Digiprep MS block digestor (SPS Science) equipped with a temperature-time programmable controller and polypropylene digestion tubes. ‘Aqua regia’ digestion does not extract those TEs associated with silicates [47]. Nevertheless, this method is widely accepted to characterize TE concentrations in soil pollution studies, because the silicate-binding metals have less environmental implications. These concentrations are referred to as ‘pseudototal’ concentrations. The quality of the analysis was assessed using the reference soil sample ERM-CC141 (Loam soil). Obtained recoveries ranged from 95.3% to 101%. Bioavailability of selected TEs was estimated by extraction with 0.01 M CaCl_2_ solution (1/10 soil/solution ratio, fraction < 2 mm) and analysis by ICP-OES [48].

### Data analyses

Differences among sites in soil and plant (leaf and flower bud) chemical composition were tested by one-way ANOVA and post-hoc Tukey tests (P < 0.05). Data normality and homogeneity of variance were verified by means of Kolmogorov–Smirnov and Levene tests, respectively. When data did not meet normality they were logarithmic transformed. In the case of Mn, even after transformation did not meet normality, therefore Kruskal–Wallis tests were used for comparison of means.

Factorial ANOVAs were applied to test for differences in TE composition between plant organs (leaves versus flower buds) and between sites, and to test for possible interactions between both factors (organ × site interaction). For Mn and As, which showed a high heterogeneity between organs and /or across sites, linear models using generalized least squares were fitted, with the nlme package in R 3.1.2. In these models, a variance structure parameter was added to account for heterogeneity of model residuals between organs and/or sites [49]. Significance level was p < 0.05.

Bivariate relationships between TE concentrations in soils and plants were explored using Spearman correlational analysis. The overall pattern of covariation of the TEs in leaves and flower buds was explored by Principal Component Analysis. In order to remove closure effects in chemical compositional data, dataset was previously transformed using the centred-log ratio transformation function of R package “rgr”. All statistical analyses were conducted using the program SPSS 23.0 for Windows.

To evaluate soil pollution severity, we used the pollution load index (PLI) as defined by Tomlinson [50]. This index is based on the concentration factor (CF) of each metal (As, Cd, Cu, Pb and Zn), which is defined as the ratio between the concentration of the metal in each soil and the background values for the region [51]. For each sampling site, PLI is calculated as the n^th^ root of the product of the obtained CFs for each element. Values of PLI close to 1 indicate heavy metal loads near the background level, while values above 1 indicate soil pollution [51].

For a general comparison background values for total concentrations (median, normal soils of the world) were taken from [52], and available concentrations from unaffected soils of SW Spain from [53]. Intervention values for Andalusian soils (total concentrations) were taken from [54], and permitted water soluble levels from [55], except the value for Mn which refers to the critical level (CaCl_2_ extracted) that can affect many plants [56].

In order to estimate what proportion of the total soil metal concentration was available and transferred to leaves and flower buds, the transfer coefficient (TC) was calculated. This is defined as the ratio of metal concentration in the plant [M]_plant_ to the total metal concentration in the soil [M]_soil_ [57]. The TC of TEs was compared between *E*. *camaldulensis* and *S*. *purpurea*.

## Results

### General soil properties and pollution levels

*E*. *camaldulensis* trees were growing (planted before the mine accident) in a wide variety of soils within the Guadiamar basin (S1 Table). Soil acidity ranged from low pH (5.6) in the rocky slopes of site C1 to the basicity (pH 8.1) of the sandy plains, site C2. Texture was also variable between sandy loam and silty clay. Fertility was higher in the sites from the southern, lower part of the river (S3, S4 and S5), having clayey texture and relatively higher values of organic carbon (13–30 g kg^-1^), nitrogen (1.2–2.5 g kg^-1^) and available K (186–473 mg kg^-1^). However, available phosphorus was low across all sites, ranging from 1 mg kg^-1^ (in deeper soils of C1, C2 and S3) to 15 mg kg^-1^ (in topsoil of S5).

The contamination within the study area was variable (S1 Fig), from cleanness (PLI below unity) in the sites non-affected by the mine spill (C1 and C2) to highly contaminated (PLI up to 11) in the deeper soil of the spill-affected and non-remediated plot (S3). Contamination increased with soil depth (except in site S2).

Total concentrations of sulphur and TEs were significantly different between the sites, at both soil depths (Table 1 and S3 Table). The greatest variability between plots for As, Pb and S occurred deeper in the soil profile (20–40 cm). Thus, the non-remediated plot S3 had the highest subsoil concentrations of arsenic (1,069 mg kg^-1^), lead (4,086 mg kg^-1^) and sulphur (53,635 mg kg^-1^), compared with the lowest values of clean sites (1.6 mg kg^-1^ As and 6 mg kg^-1^ Pb in site C2, and 65 mg kg^-1^ S in site C1). Both As and Pb maximum values surpassed the Andalusian intervention thresholds (Table 1), [54].

**Table 1:** Comparative analysis of total (‘aqua regia’) and CaCl_2_-extractable concentrations (mg kg^-1^) of S and six trace elements in soils where *E*. *camaldulensis* trees were sampled in the Guadiamar Basin.

Element	*Surface soil (0–20 cm)*				Deep soil (20–40 cm)				Background values	Intervention values
	Mean	Range	F	p	Mean	Range	F	p		
*Total*										
As	81.1	2.37–265	25.20	<0.001	256	1.60–1069	49.58	<0.001	6	>50
Cd	0.97	0.01–2.79	15.89	<0.001	1.23	0.01–3.65	13.22	<0.001	0.35	>10
Cu	74.6	5.74–223	9.71	<0.001	105	4.31–241	10.96	<0.001	30	>500
Mn	581	123–1477	20.99	<0.001	505	113–1705	12.67	<0.001	1000	-
Pb	362	9.54–4383	14.15	<0.001	635	5.87–4086	27.92	<0.001	35	>500
S	2568	105–17650	23.70	<0.001	6763	65.4–53635	57.72	<0.001	700	-
Zn	308	13.6–742	13.12	<0.001	417	11.3–1218	13.60	<0.001	90	>1000
Extractable										(permitted water soluble levels)
As	<dl	<dl	<dl	<dl	<dl	<dl	<dl	<dl	<dl	0.04
Cd	0.02	0.001–0.11	12.78	<0.001	0.02	0.004–0.14	9.49	<0.001	0.001	0.03
Cu	0.23	0.11–0.47	15.29	<0.001	0.24	0.08–0.88	5.25	0.005	0.01	0.70
Mn	4.64	0.20–25.8	37.22	<0.001	4.12	0.09–20.5	14.45	<0.001	0.08	> 65
Pb	0.05	0.00–0.59	8.708	0.191	0.10	0.001–0.75	6.63	0.356	<dl	1.0
S	668	0.96–5068	39.38	<0.001	1231	0.25–5342	10.72	<0.001	8.44	-
Zn	0.86	0.09–5.54	17.52	0.008	1.31	0.10–13.8	15.03	0.020	0.22	0.5

Mean (N = 3) and range values, and results of the factorial ANOVA (F-parameter) applied; with exception of available Pb and Zn where Kruskal–Wallis (H parameter) was used. Significance values (p-values) are indicated (p ≤ 0.05). Abbreviaton dl means detection limit.

The pattern of variation in the TE concentration at the top 20cm were similar to the deeper layer, with TE concentrations being in general lower in the 0-20cm layer. The maximum concentrations in topsoil of As, Cd, Pb and S were found at the non-remediated plot S3. Other elements varied in the spatial contamination patterns of topsoil, Cu had the maximum value (223 mg kg^-1^) in site S2, Mn had its maximum (1,477 mg kg^-1^) in site S1 and Zn peaked (742 mg kg^-1^) at site S5 (S3 Table).

The extractability of TEs (CaCl_2_ extraction) was generally two orders of magnitude lower than total values. In the case of As, extractable values were below detection limits. There were significant differences between sites in the extractability of TEs, with exception of Pb (Table 1). The most variable elements (with highest F values) were S (ranging from 1 to 5,068 mg kg^-1^) and Mn (from 0.2 to 26 mg kg^-1^) in the topsoil (S4 Table). Availabilities of Cd and Zn were relatively high, and clearly surpassed the permitted levels (for Andalusian soils) [55]: maximum of 0.14 mg kg^-1^ Cd above the critical 0.03 mg kg^-1^ level, and maximum of 14 mg kg^-1^ Zn above the permitted 0.5 level (Table 1).

### Chemical composition of leaves and flower buds

Concentrations of the 12 studied elements in leaves and flower buds of *E*. *camaldulensis* differed significantly between sites (Table 2; for more details see S5 and S6 Tables). The greatest inter-site differences (highest F values) were found for Zn, Cu and As, and the lowest for the nutrients Mg, Ca and S. With the exception of K, element concentrations in leaves and flower buds differed significantly between sites. The highest between-organ differences were found for the non-essential and toxic elements As and Cd. There were significant site-organ interactions for seven out of 12 elements.

**Table 2:** Results of the factorial ANOVA (F-parameter and p-values) applied to *E*. *camaldulensis* chemical composition, with site and plant organ as explaining factors.

Element	Site		Organ		Site x Organ	
	F	p	F	p	F	p
**As**	20.96	<0.0001	326.14	<0.0001	35.87	<0.0001
**Ca**	3.57	0.009	54.10	<0.0001	2.13	0.081
**Cd**	10.34	<0.0001	362.30	<0.0001	2.11	0.084
**Cu**	22.60	<0.0001	6.65	0.015	3.16	0.017
**K**	16.20	<0.0001	2.40	0.132	2.84	0.027
**Mg**	3.16	0.017	87.30	<0.0001	2.66	0.036
**Mn**	15.37	<0.0001	22.83	0.001	1.97	0.104
**N**	8.79	<0.0001	180.40	<0.0001	2.71	0.034
**P**	6.84	<0.0001	9.19	0.005	2.64	0.037
**Pb**	5.99	<0.0001	94.90	<0.0001	1.67	0.165
**S**	3.97	<0.0001	221.80	<0.0001	0.63	0.704
**Zn**	34.30	<0.0001	90.01	<0.0001	4.36	0.003

The significance level was p ≤ 0.05.

**Fig 3:**
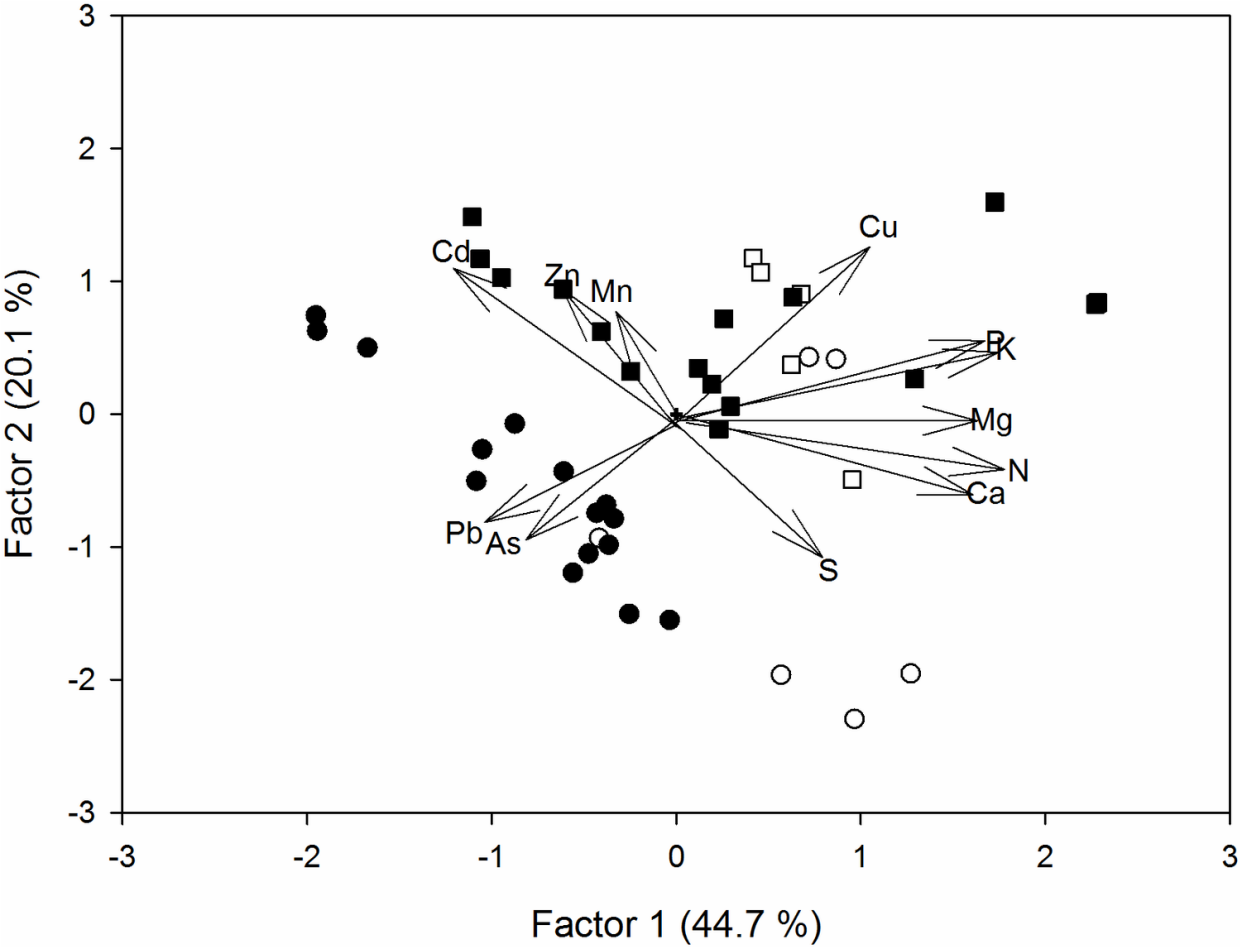
Results of the principal component analysis (PCA) of *E*. *camaldulensis* chemical composition. Symbols are circles for leaves and squares for flower buds; those sampled in contaminated sites are indicated in black and those in clean sites in white. Vectors represent the eigenvector coefficients (multiplied by two, for clarity) of the 12 elements.

The general variation in the chemical composition, between sites and between leaves and flowers, is shown in Fig 3. The first axis of the Principal Component Analysis (PCA) explained the 44.7% of the variance and was mainly defined positively by the concentrations of nutrients N, Ca, K, Mg and P, and negatively by Cd, Pb and As. There was a general trend of highest trace element accumulation in leaves from contaminated sites. The second PCA axis explained 20.1% of the variance and was related to Cu, Cd and Zn (positive), and S (negative) concentrations.

For a more detailed analysis of the differences among sites and between flowers and leaves, we have separated macronutrients (N, P, S, K, Ca and Mg) and TEs (As, Cd, Cu, Mn, Pb and Zn).

**Fig 4:**
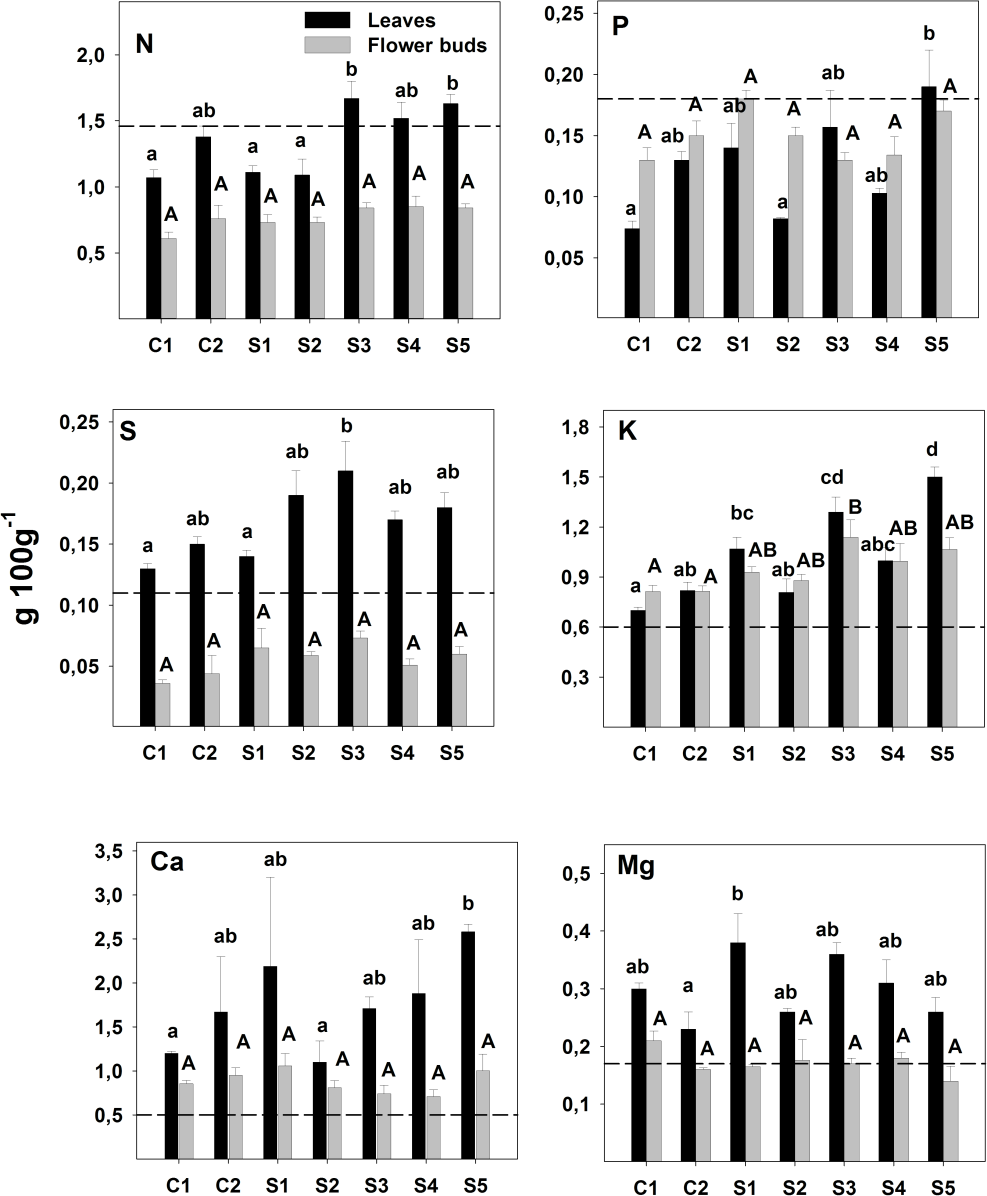
Nutrient concentrations in *E*. *camaldulensis* leaves and flower buds (mean ± standard error). Significant differences (p < 0.05) among sites for leaves composition (black bars) are marked with lower case letter, while difference for flowers buds (grey bars) are in capital letters. As reference for each nutrient, a dotted line indicates the lower limit of the range considered adequate for *E*. *camaldulensis* normal growth [58].

Macronutrient concentrations were greater in leaves than in flower buds, except for P and K. *E*. *camaldulensis* foliar levels of S, K, Ca and Mg were above the limit considered adequate for normal growth in all the seven sites [58]. However, N and P values could indicate some nutrient deficiency (Fig 4).

**Fig 5:**
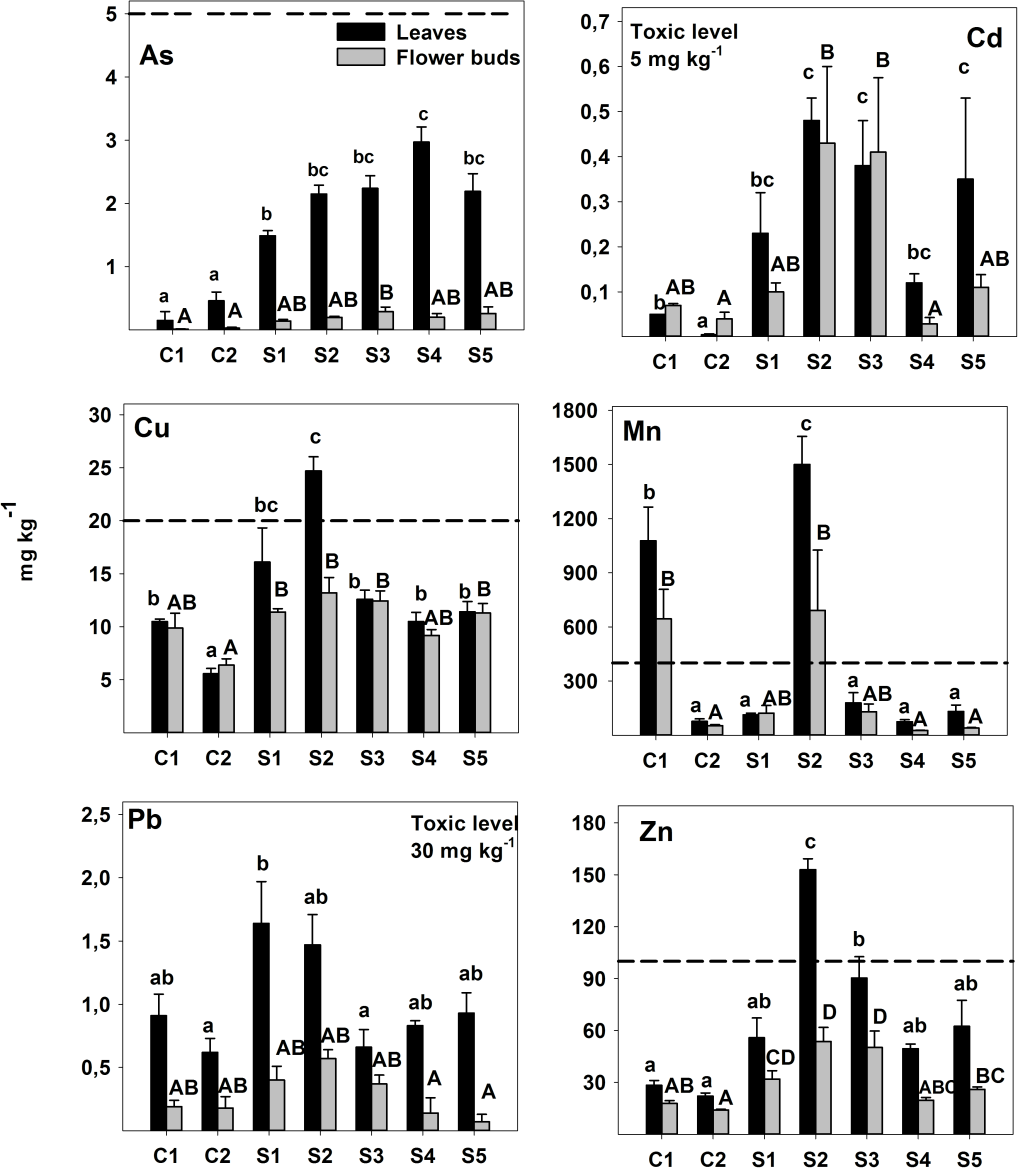
Trace element concentrations in *E*. *camaldulensis* leaves and flower buds (mean ± standard error). Significant differences (p < 0.05) among sites for leaves composition (black bars) are marked with lower case letter, while difference for flowers buds (grey bars) are in capital letters. As reference for TE, a dotted line indicates the lower limit of the range considered toxic for plants [59].

As expected, the concentration of TEs in *E*. *camaldulensis* leaves and flower buds were significantly lower in the clean sites, in particular in site C2, than in those affected by the mine spill. Flower buds tended to accumulate lower concentrations than leaves, especially of the non-essential elements As and Pb (Fig 5).

The contaminated site S2 was remarkable for the highest values of TEs in leaves, in particular of Zn, Cu and Mn, above the phytotoxicity levels [59]. The case of Mn, which had high foliar values in trees from non-affected rocky slopes (site C1), could indicate a geological origin. Other TEs of known toxicity like As, Cd and Pb were accumulated at safe levels.

### Soil and plant relations

The transfer coefficients (TC) from soil to leaves indicate the accumulation (>1) or exclusion (<1) of the particular TE by *E*. *camaldulensis*. In most cases, the transfer was low and TEs present in soil were not accumulated in *E*. *camaldulensis* leaves. The relevant exception was the high accumulation of Mn in site S2 (TC of 4–5). The relatively high TC values obtained in non-affected sites (Cd in site C1 and Zn in site C2) correspond to low absolute values in both soil and leaves and therefore of lower importance. Arsenic and Pb showed the lowest coefficients, indicating a limited transfer of both elements from soils to *E*. *camaldulensis* leaves (Table 3).

**Table 3:** Transfer coefficients (TC) of six trace elements from soil (total concentrations, at two depths) to leaves at seven sampling sites for *E*. *camaldulensis* and comparative values for *S*. *purpurea* (at two sites).

Species	Site	As		Cd		Cu		Mn		Pb		Zn	
		0–20 cm	20–40 cm	0–20 cm	20–40 cm	0–20 cm	20–40 cm	0–20 cm	20–40 cm	0–20 cm	20–40 cm	0–20 cm	20–40 cm
		^_________________________________________________________________________________________________________________________________^		^_________________________________________________________________________________________________________________________________^		^_________________________________________________________________________________________________________________________________^		^_________________________________________________________________________________________________________________________________^		^_________________________________________________________________________________________________________________________________^		^_________________________________________________________________________________________________________________________________^	
*Eucalyptus*	C1	0.01	0.01	**2.68**	**1.51**	0.29	0.32	**1.04**	**1.04**	0.03	0.03	0.39	0.37
*camaldulensis*	C2	0.17	0.24	0.63	0.27	0.63	1.03	0.53	0.61	0.06	0.09	1.28	1.65
	S1	0.05	0.04	0.49	0.21	0.32	0.25	0.12	0.17	0.02	0.02	0.31	0.18
	S2	0.02	0.03	0.86	**1.28**	0.18	0.22	**3.85**	**4.83**	0.007	0.008	0.62	0.83
	S3	0.01	0.002	0.25	0.35	0.14	0.08	0.42	0.85	0.001	0.0003	0.19	0.21
	S4	0.03	0.01	0.12	0.06	0.15	0.08	0.17	0.16	0.005	0.002	0.16	0.08
	S5	0.03	0.01	0.15	0.14	0.11	0.06	0.27	0.31	0.008	0.002	0.10	0.07
*Salix*	S1	0.14	0.11	**6.67**	**2.80**	0.19	0.15	0.53	0.83	0.03	0.02	**2.60**	**1.52**
*purpurea*	S2	0.05	0.05	**10.5**	**19.4**	0.06	0.08	0.67	0.74	0.01	0.01	**3.47**	**5.17**

Values higher than unity (indicating accumulation) are marked in bold.

The comparative values of TC for *S*. *purpurea* reflected a high transfer and accumulation of Cd and Zn in willow leaves, with maximum of 19 for Cd and 5 for Zn (in site S2). The concentration of Cd in leaves of *S*. *purpurea* (mean of 7 mg kg^-1^ in site S2) was about 15 times higher than in *E*. *camaldulensis*, and above the phytotoxicity threshold (5 mg kg^-1^). Similarly, Zn concentration in willow leaves was about six times higher than in *E*. *camaldulensis*, both surpassing the phytotoxicity level (100 mg kg^-1^) in site S2 (S7 Table).

The concentration of a determined TE in the *E*. *camaldulensis* leaves can be used as bioindicator if it is significantly correlated with its concentration in soil. Thus Cd, Mn and Zn levels in leaves indicated (i.e., they were significantly correlated with) the available concentration of that TE in soil, at the two depths (Table 4). Leaf As was also correlated, but with total concentration of As in soil (extractable concentration was below detection limit and correlation could not be calculated). However, the correlation leaf-soil was not significant for Cu and Pb, neither for total nor extractable soil values.

**Table 4:** Correlation coefficients between available trace elements in soil (CaCl_2_ -extracted) and the concentration of the same element in *E*. *camaldulensis* leaves and flower buds.

Element	Organ	Soil depth	Correlation coefficient
**As**	Leaves	0–20 cm	0.792**
		20–40 cm	0.792**
	Flower buds	0–20 cm	0.743**
		20–40 cm	0.803**
**Cd**	Leaves	0–20 cm	0.690**
		20–40 cm	0.748**
	Flower buds	0–20 cm	0.588**
		20–40 cm	0.606**
**Cu**	Leaves	0–20 cm	0.114
		20–40 cm	0.297
	Flower buds	0–20 cm	0.225
		20–40 cm	0.109
**Mn**	Leaves	0–20 cm	0.614**
		20–40 cm	0.621**
	Flower buds	0–20 cm	0.604**
		20–40 cm	0.722**
**Pb**	Leaves	0–20 cm	0.067
		20–40 cm	-0.210
	Flower buds	0–20 cm	0.050
		20–40 cm	0.065
**Zn**	Leaves	0–20 cm	0.517*
		20–40 cm	0.555**
	Flower buds	0–20 cm	0.422
		20–40 cm	0.536*

Significance levels are

**p<0.01

*p < 0.05.

Arsenic correlations were calculated using pseudototal contents in soils.

## Discussion

### Heterogeneity of soil conditions

*Eucalyptus camaldulensis* is a generalist tree species, which can grow in a variety of soil conditions [23, 28, 60]. In the study area, *E*. *camaldulensis* demonstrated the capacity to grow in soils with contrasting pH (values from 5.6 to 8.1) and texture (sandy to clayey). They thrived on impoverished soils, in particular with organic N concentration below 1 g kg^-1^, a value considered as low for most soils in the Southern Spain. In most cases, the soil C/N ratio was > 15, which can be indicative of a very low N availability [61].

Levels of P availability were also low [61]. However, K concentrations were at ‘critical’ or ‘low’ levels in the sandy soils, especially at the deep soil, and normal, or even high levels in the more silty-clayey soils. In general, the organic matter content surpassed the critical value of 20 g kg^-1^ (see [61] for critical and low values of soil nutrients, and [62] for general overview of soil fertility of the study area).

The study area was contaminated by a mine-spill on 1998, and despite the soil cleaning and remediation operations, there was still residual contamination (18 years later), in particular in the riparian areas [7]. Soil samples taken from the *E*. *camaldulensis* rhizosphere had high total concentrations of some TEs. In particular, As reached up to 765 mg kg^-1^ (at site S5) well above the Andalusian intervention value of 50 mg kg^-1^, and Pb had maximum values of 1790 mg kg^-1^ (site S5) and 1189 mg kg^-1^ (site S4) surpassing the intervention value of 500 mg kg^-1^ [54]. Soil acidity would increase the bioavailability of trace elements and therefore their potential toxicity [57].

The total S concentration in soil was high (up to 17501 mg kg^-1^ at S5) reflecting the pyritic origin and S-enrichment of the mine sludge [51]. The highest values of total Mn in soil were found at the non-contaminated rocky site (C1; up to 1705 mg kg^-1^) and is likely of geologic origin; however that natural concentration is >500 mg kg^-1^, a limit for which ecological investigation has been recommended [63].

### Variability in chemical composition of leaves and flower buds

The concentration of mineral elements in *E*. *camaldulensis* leaves can indicate the nutrient status of the tree as well as to detect potential toxicity by TEs ([58, 59, 64]. Nitrogen and P had relatively low concentrations in the studied *E*. *camaldulensis* trees indicating some deficiency. While the levels of K, Ca, Mg and S in leaves were adequate for a normal growth of this species [58]. The relatively high concentration of Ca in leaves can help the trees to tolerate the high concentration of TEs in the soil solution, due to the potential protective action of Ca against the toxicity of metals and metalloids [65, 66].

Despite the high total concentration of As and Pb in soils, the *E*. *camaldulensis* leaves accumulated relatively low amounts of those elements, with values below the phytotoxicity level. However, the maximum values of Cu, Mn and Zn measured in leaves of some trees (site S2) reached phytotoxic levels. At that particular site, the soil was acidic (pH 6.3) and low in organic C (8.5 g kg^-1^), thereby increasing the availability of TEs. Both factors, especially pH, are key determinants of the bioavailability of trace elements in soil [59]. In addition, trees were growing close to the river and therefore maintaining high transpiration rates and high transfer rates of TEs from deep soil through the deep sinker roots typical of *Eucalyptus* species [67]. Nevertheless, those high levels of Cu, Mn and Zn found at that particular site do not necessarily imply a toxic response, because the so-called “phytotoxic ranges” are illustrative guidelines that may vary across species, depending on specific adaptations [59].

The high accumulation of Mn into leaves seems to be a particular feature of *Eucalyptus* species, which has been previously reported [68–70], probably associated with a high tolerance of that element. The highest values of leaf Mn reflected the Mn-enrichment of soils and apparently were of geologic origin, independent of the mine-spill. The acidity of soils contributed to the Mn solubilization in the soil matrix [59, 71]. The maximum value of Mn measured here for *E*. *camaldulensis* leaves (1772 mg kg^-1^) can be compared with those found for other species, such as *E*. *globulus* (4000 mg kg^-1^; [70]) and the hybrid *E*. *grandis* × *E*. *urophylla* (13500 mg kg^-1^) [69].

Flower buds had a significantly different chemical composition than leaves, with exception of potassium. Transport of mineral elements differs among leaves and reproductive structures; for instance, flowers and fruits usually have weak xylem but strong phloem translocation activities [72]. Non-essential elements, such as As and Pb, had lower concentrations in the flower buds, probably reflecting the action of a physiological barrier impeding their translocation to the seeds [73, 74]. However, there are some studies showing a high accumulation of TEs in flowers and seeds [75, 76]. In addition, metals and metalloids can be transferred to pollen grains and nectar, potentially affecting the composition of apiary products [31].

#### Potential for phytostabilization

Trees within the *Eucalyptus* genus present many of the features to be selected for phytostabilization in Mediterranean environments: tolerance to high concentrations of contaminants, ability to develop an extensive root system, low maintenance requirements, relatively high transpiration rates, relatively long growing period, ability for self-propagation, and adaptation to local climate [77]. Despite the low concentrations of soil nutrients and the relatively high concentration of some TEs (As, Pb and Mn) *E*. *camaldulensis* grew without toxicity symptoms in all the sites of the study area. They showed plasticity in tree growth and leaf morphology along the Guadiamar basin (S2 Table). In the soils with higher fertility (lower part of the river, sites S4 and S5), trees were taller and with larger trunks; leaves of lower LMA indicated higher relative growth rate [39]. On the contrary, trees on drier slopes (site C1) were smaller and showed water stress (lower absolute values of δ^13^C) [78].

Phytostabilization aims to immobilize TEs within the plant rhizosphere, and thus reducing their bioavailability and minimizing the exposure of livestock, wildlife, and human populations to them [79]. To be suitable for phytostabilization, the transfer of TEs from soil to aboveground plant organs must be low (TC<1) [80]. Our results show that *E*. *camaldulensis* is appropriate for the phytoestabilization of soils contaminated by Cd, As and Pb; given the relatively low levels of accumulation of these elements recorded in the aboveground biomass. Previous studies have also highlighted the ability of this and other *Eucalyptus* species for the phytostabilization of TEs on contaminated soils [28, 81, 82]. In contrast, the studied willow (*Salix purpurea*) accumulated high concentrations of foliar Cd (up to 10.9 mg kg^-1^) and Zn (maximum of 1126 mg kg^-1^); therefore this native species, which has naturally revegetated the riparian area, would not be suitable for planting and phytostabilizing contaminated sites.

Manganese is an essential element but in high concentration can induce some toxicity; e.g., a threshold of 1370 mg g^-1^ of Mn in the diet reduced the survival of moth larvae [83]. Although *Eucalyptus* leaves are rarely consumed outside its native habitat, some phytophagous insects feeding on them could, in turn, provide a Mn-rich diet for birds [84].

The assessment of the phytostabilization ability of a plant species is usually limited to the analysis of metal accumulation into foliage. However, other organs that may be consumed by herbivores or, indirectly, by humans should also be investigated. *Eucalyptus* honey is commonly produced for human consumption [85, 86]. We found that the flower buds effectively excluded As and Pb, and that Cd concentration was below the threshold value for human consumption (0.5 mg kg^-1^ dw, based on [87]). This indicates that the concentrations of these elements in nectar and therefore potential toxicity are likely to be low. However, further analyses of nectar, pollen and honey, in particular of the accumulating Mn, are needed to confirm its safety for human consumption.

The banks and channels of the Guadiamar River have acid soils and residual contamination, originated from the mine-spill. Appropriate management should include application of soil amendments for pH correction, and revegetation of the riverbanks to ensure mechanical stabilization, and to reduce soil losses through water erosion [7]. Existing *E*. *camaldulensis* trees can play a role in soil phytostabilization of this fragile ecosystem.

#### Potential for biomonitoring of soil pollution

Biomonitors are defined as organisms that contain information on the quantitative aspects of the quality of the environment [10]. For most plants, except metal excluders [88], the accumulation of a TE in a particular plant organ confirms its availability in the soil. The general advantages of biomonitoring are: the permanent and common occurrence of an organism in the field, even in remote areas, the ease of sampling, and the absence of any necessary expensive technical equipment [9, 12]. Thus, biomonitors can be used to detect low concentrations that are not always easy to measure directly using chemical extraction techniques; even if they are measurable as total levels, their ecological relevance is often difficult to determine from soil concentrations. As far as we know, there is no information about the potential use of eucalyptus, widely planted in all types of soil, for biomonitoring soil pollution.

*E*. *camaldulensis* leaves were good indicators (with high correlation values) of soil concentration for As, Cd, Mn and Zn (Table 4). Manganese in flower buds also had a remarkably high correlation with extractable Mn in soil. Although Mn uptake is metabolically controlled, passive absorption is also likely to occur, especially when concentrations in the soil solution are high and within the phytotoxic range [59]. The results suggest that on the Guadiamar study area *E*. *camaldulensis* leaves and flower buds could be used to biomonitor Mn availability in soil.

Potential biomonitoring of Cd and Zn in soils is relatively common in plants because these elements are passively absorbed through the roots and freely translocated to leaves and other plant organs [89]. Thus, *E*. *camaldulensis* leaves are also biondicators of soil Cd and Zn in the Guadiamar soils. In previous studies, white poplar (*Populus alba*), a native tree in the riparian Guadiamar forests, has been proposed for bioindication of soil Cd and Zn [11, 12].

In contrast, the weakness of the relationships between soil and plant concentrations for Cu and Pb is likely due to the high effectivity of the retention of these elements in plant roots [59]. Therefore *E*. *camaldulensis* leaves are not bioindicators for soil Cu and Pb.

## Conclusions

*E*. *camaldulensis* is a good candidate for the phytostabilization and biomonitoring of TE contaminated soils because of its tolerance to a wide variety of soil conditions, rapid growth rate, and low rates of herbivory. Despite the high soil concentration of some toxic TEs, such as As and Pb (surpassing the intervention values), their accumulation in leaves of *E*. *camaldulensis* were relatively low, and below toxic levels. The exception was Mn, which accumulated in *E*. *camaldulensis* leaves in two sites, apparently from geologic origin and independent of the spill contamination, but it should require some specific monitoring and caution. In general, the accumulation of TEs in flower buds was even lower than in leaves. Although the general concentration of TEs in leaves was low, there were some significant relationships with the soil extractability of Cd, Mn and Zn; therefore, *E*. *camaldulensis* is valuable for biomonitoring of those three elements.

## Supporting information

S1 Fig Values of pollution load index (PLI) for two soil depths at the seven sites sampled in the Guadiamar River valley (SW Spain). (DOCX)

S1 Table General soil parameters analyzed at two depths (0–20 and 20-40cm) of the seven sampling sites with specific location indicated below. Mean values ± SE; n = 3 except for N, Org. C and texture with a single value from a composite sample.
(DOCX)

S2 Table Allometric measures of *E*. *camaldulensis* trees in each sampling site (mean values ± SE; n = 3). Tree height, trunk diameter at breast height (DBH), leaf mass per area (LMA) and leaf concentration of ^13^C.
(DOCX)

S3 Table Total concentration (mean ±SE) of nine TEs at the seven study sites of the Guadiamar Valley. Maximum and minimum values are indicated between brackets.
(DOCX)

S4 Table Available concentrations of S and five trace elements at each sampling site (mg kg^-1^; mean values ± SE). Range for each element in parenthesis. Abbreviation dl means detection limit.
(DOCX)

S5 Table Macronutrient concentrations in leaves and flower buds at each sampling site (g 100g^-1^; mean values ± SE). Range for each element in parenthesis.
(DOCX)

S6 Table Trace element concentrations in leaves and flower buds at each sampling site (mg kg^-1^; mean values ± SE). Range for each element in parenthesis.
(DOCX)

S7 Table Cadmium and Zn concentrations (mg kg^-1^) of *Eucalyptus camaldulensis* and *Salix viminalix* at sites S1 and S2. Significant differences per species at each site and organ are marked with and asterisk (p<0.05).
(DOCX)
