# Supplementary material for: Potential of Eucalyptus camaldulensis for phytostabilization and biomonitoring of trace-element contaminated soils
Source: PLoS One. 2017 Jun 30;12(6):e0180240. doi: 10.1371/journal.pone.0180240 (PMC5493371; doi:10.1371/journal.pone.0180240)
Supplement: S1 Fig — (DOCX) [file pone.0180240.s001.docx]

**SUPPORTING INFORMATION**

**S1 Figure**. Values of pollution load index (PLI) for two soil depths at the seven sites sampled in the Guadiamar River valley (SW Spain).
