# Supplementary material for: Potential of Eucalyptus camaldulensis for phytostabilization and biomonitoring of trace-element contaminated soils
Source: PLoS One. 2017 Jun 30;12(6):e0180240. doi: 10.1371/journal.pone.0180240 (PMC5493371; doi:10.1371/journal.pone.0180240)
Supplement: S1 Table — Mean values ± SE; n = 3 except for N, Org. C and texture with a single value from a composite sample. (DOCX) [file pone.0180240.s002.docx]

**S1 Table.** Geographical coordinates and general soil parameters in the seven sampling sites, analyzed at two depths (0-20 and 20-40cm). Mean values ± SE; n=3 except for N, Org. C and texture, with a single value from a composite sample.

| Site | Latitude  Longitude | Depth  (cm) | pH | CaCO_3_  (g kg^-1^) | Org. C  (g kg^-1^) | N  (g kg^-1^) | P  (mg kg^-1^) | K  (mg kg^-1^) | Texture |
| --- | --- | --- | --- | --- | --- | --- | --- | --- | --- |
| C1 | 37º31.805’ N  6º16.823’ W | 0-20 | 5.6 ± 0.1 | 0.1 ± 0.0 | 18.6 | 1.85 | 2.4 ± 0.1 | 141 ± 26.0 | Loam |
|  |  | 20-40 | 5.6 ± 0.2 | 0.1±0.0 | 5.0 | 0.90 | 1.0 ± 0.1 | 59.3 ± 14.5 | Loam |
| C2 | 37º13.679’ N  6º11.288’ W | 0-20 | 7.9 ± 0.0 | 2.5 ± 0.7 | 15.1 | 0.98 | 4.8 ± 0.6 | 132 ± 14.4 | Sandy loam |
|  |  | 20-40 | 8.1 ± 0.0 | 2.7 ± 0.4 | 2.6 | 0.41 | 1.4 ± 0.3 | 108 ± 16.8 | Sandy loam |
| S1 | 37º29.298’ N  6º13.213’ W | 0-20 | 7.8 ± 0.1 | 2.8 ± 0.4 | 15.3 | 1.09 | 10.2 ± 1.4 | 183 ± 13.0 | Sandy loam |
|  |  | 20-40 | 7.8 ± 0.0 | 2.5 ± 0.4 | 13.0 | 0.88 | 4.5 ± 0.4 | 157 ± 18.7 | Sandy loam |
| S2 | 37º27.975’ N  6º12.712’ W | 0-20 | 6.3 ± 0.4 | 0.1 ± 0.0 | 8.5 | 0.61 | 3.7 ± 0.3 | 79.7 ± 8.99 | Loamy sand |
|  |  | 20-40 | 6.7 ± 0.0 | 0.3 ± 0.2 | 5.7 | 0.43 | 2.0 ± 0.8 | 60.7 ± 9.90 | Loamy sand |
| S3 | 37º22.505’ N  6º13.484’ W | 0-20 | 6.7 ± 0.1 | 9.1 ± 1.6 | 27.2 | 2.24 | 9.3 ± 1.6 | 392 ± 40.7 | Silty clay |
|  |  | 20-40 | 6.0 ± 0.2 | 3.7 ± 0.8 | 13.0 | 1.23 | 0.9 ± 0.0 | 186 ± 47.4 | Silty clay |
| S4 | 37º17.591’ N  6º15.968’ W | 0-20 | 7.3 ± 0.1 | 11.9 ± 1.1 | 30.1 | 2.52 | 10.9 ± 2.1 | 473 ± 32.6 | Silty clay |
|  |  | 20-40 | 6.8 ± 0.2 | 9.1 ± 1.7 | 12.9 | 1.64 | 3.8 ± 1.3 | 318 ± 26.9 | Silty clay |
| S5 | 37º15.815’ N  6º15.834’ W | 0-20 | 7.3 ± 0.1 | 11.7 ± 0.4 | 23.7 | 2.32 | 15.4 ± 3.4 | 359 ± 43.6 | Clay loam |
|  |  | 20-40 | 6.9 ± 0.2 | 8.1 ± 2.6 | 12.7 | 1.54 | 6.3 ± 3.4 | 222 ± 29.4 | Clay loam |
