# Supplementary material for: Potential of Eucalyptus camaldulensis for phytostabilization and biomonitoring of trace-element contaminated soils
Source: PLoS One. 2017 Jun 30;12(6):e0180240. doi: 10.1371/journal.pone.0180240 (PMC5493371; doi:10.1371/journal.pone.0180240)
Supplement: S2 Table — Tree height, trunk diameter at breast height (DBH), leaf mass per area (LMA) and leaf concentration of 13C. (DOCX) [file pone.0180240.s003.docx]

**S2 Table**. Allometric measures of *E. camaldulensis* trees in each sampling site (mean values ± SE; n=3). Tree height, trunk diameter at breast height (DBH), leaf mass per area (LMA) and leaf concentration of ^13^C. Tree ring analyses (at 1.3 m height) resulted in approximately 21 years for C1, 19 years for S1 and 12 years for S2 (by dendrometric analysis; Raúl Sánchez-Salguero, pers. comm.)

| Site | Height  (m) | DBH  (cm) | LMA  (kg m^-2^) | δ^13^C  (‰) |
| --- | --- | --- | --- | --- |
| C1 | 16.1 ± 0.9 | 43.0 ± 6.7 | 0.203 ± 0.007 | -27.16 ± 0.06 |
| C2 | 20.5 ± 1.5 | 31.1 ± 1.7 | 0.152 ± 0.011 | -29.35 ± 0.53 |
| S1 | 16.6 ± 2.1 | 31.7 ± 5.1 | 0.132 ± 0.002 | -30.40 ± 0.05 |
| S2 | 13.9 ± 0.7 | 29.4 ± 2.8 | 0.166 ± 0.002 | -28.79 ± 0.50 |
| S3 | 20.9 ± 1.9 | 34.0 ± 3.4 | 0.118 ± 0.023 | -30.62 ± 0.56 |
| S4 | 25.1 ± 1.5 | 47.3 ± 7.7 | 0.107 ± 0.004 | -29.48 ± 0.13 |
| S5 | 26.5 ± 1.3 | 50.2 ± 1.6 | 0.098 ± 0.012 | -31.47 ± 0.73 |
