# Supplementary material for: Potential of Eucalyptus camaldulensis for phytostabilization and biomonitoring of trace-element contaminated soils
Source: PLoS One. 2017 Jun 30;12(6):e0180240. doi: 10.1371/journal.pone.0180240 (PMC5493371; doi:10.1371/journal.pone.0180240)
Supplement: S3 Table — Maximum and minimum values are indicated between brackets. (DOCX) [file pone.0180240.s004.docx]

**S3 Table**. Total concentrations of S and six trace elements at each sampling site (mg kg^-1^; mean values ± SE). Range for each element in parenthesis.

| Site | Depth | As | Cd | Cu | Mn | Pb | S | Zn |
| --- | --- | --- | --- | --- | --- | --- | --- | --- |
| C1 | 0-20 | 18.2 ± 5.63  (11.6-29.4) | 0.03 ± 0.01  (0.01-0.04) | 37.2 ± 2.44  (33.4-41.7) | 1046 ± 185  (688-1304) | 32.2 ± 0.47  (31.4-32.9) | 170 ± 40.3  (105-244) | 73.0 ± 2.70  (67.8-76.7) |
|  | 20-40 | 19.2 ± 7.34  (10.1-34.3) | 0.08 ± 0.03  (0.01-0.13) | 33.2 ± 2.79  (30.2-38.2) | 1178 ± 344  (53-1705) | 27.6 ± 2.75  (22.6-32.0) | 83.5 ± 14.3  (65.4-112) | 77.1 ± 5.11  (68.4-86.1) |
| C2 | 0-20 | 2.97 ± 0.41  (2.37-3.77) | 0.02 ± 0.01  (0.01-0.03) | 10.2 ± 2.53  (5.74-14.5) | 151 ± 16.8  (123-181) | 11.0 ± 1.45  (9.55-13.9) | 135 ± 15.2  (115-164) | 17.8 ± 2.49  (13.5-22.2) |
|  | 20-40 | 1.97 ± 0.22  (1.60-2.37) | 0.02 ± 0.01  (0.02-0.03) | 5.52 ± 0.61  (4.31-6.24) | 127 ± 7.12  (113-135) | 7.36 ± 0.75  (5.87-8.27) | 85.5 ± 15.0  (69.9-115) | 13.4 ± 1.05  (11.3-14.5) |
| S1 | 0-20 | 28.3 ± 1.08  (26.1-29.6) | 0.63 ± 0.38  (0.23-1.39) | 50.6 ± 9.53  (39.0-69.5) | 1057 ± 231  (679-1477) | 79.6 ± 8.34  (65.9 -94.7) | 241 ± 28.0  (191-287) | 223 ± 98.7  (119-420) |
|  | 20-40 | 37.4 ± 4.77  (27.9-43.2) | 1.54 ± 0.98  (0.54-6.50) | 69.4 ± 20.4  (43.2 -110) | 713 ± 101  (519-860) | 96.1 ± 9.55  (77.0 ± 107) | 284 ± 38.8  (207-333) | 471 ± 281  (182-1034) |
| S2 | 0-20 | 127 ± 37.7  (52.1-174) | 0.79 ± 0.38  (0.39-1.54) | 154 ± 36.5  (99.0-223) | 418 ± 56.6  (306-484) | 245 ± 69.8  (112-349) | 1753 ± 666  (432-2562) | 328 ± 133  (166-592) |
|  | 20-40 | 87.4 ± 13.3  (62.4-108) | 0.48 ± 0.13  (0.24-0.68) | 125 ± 31.0  (88.8-187) | 369 ± 117  (243-302) | 215 ± 72.6  (95.4-346) | 1953 ± 184  (1594-2202) | 201 ± 34.9  (132-240) |
| S3 | 0-20 | 185 ± 49.7  (94.3-265) | 1.90 ± 0.49  (1.11-2.79) | 92.7 ± 10.8  (77.2-114) | 458 ± 44.6  (404-546) | 1804 ± 1297  (274-4382) | 10871 ± 3704  (4895-17650) | 507 ± 99.9  (345-689) |
|  | 20-40 | 985 ± 45.0  (915-1069) | 1.25 ± 0.23  (0.95-1.69) | 170 ± 21.7  (141-212) | 203 ± 31.4  (141-244) | 2709 ± 884  (1060-4086) | 30760 ± 11796  (14328-53635) | 473 ± 92.8  (342-652) |
| S4 | 0-20 | 108 ± 16.1  (86.5-139) | 1.08 ± 0.15  (0.84-1.34) | 73.0 ± 8.29  (56.8-83.9) | 444 ± 6.81  (430-453) | 180 ± 28.7b  (131-230) | 3038 ± 629  (1789-3798) | 342 ± 57.9  (242-443) |
|  | 20-40 | 276 ± 102  (111-462) | 2.07 ± 0.19  (1.75-2.42) | 143 ± 23.1  (99.8-179) | 459 ± 5.62  (451-470) | 571 ± 311  (195-1189) | 5841 ± 1399  (3423-8269) | 636 ± 56.8  (531-726) |
| S5 | 0-20 | 98.6 ± 46.8  (42.4-192) | 2.37 ± 0.06  (2.26-2.44) | 105 ± 3.98  (98.5-112) | 497 ± 41.0  (415-543) | 185 ± 78.7  (70.8-336) | 1767 ± 682  (752-3062) | 666 ± 42.2  (596-742) |
|  | 20-40 | 383 ± 193  (141-765) | 3.14 ± 0.80  (2.21-3.65) | 191 ± 27.7  (145-241) | 484 ± 72.2  (350-598) | 822 ± 485  (280-1790) | 8331 ± 4648  (2420-17501) | 1046 ± 132  (788-1218) |
