# Supplementary material for: Potential of Eucalyptus camaldulensis for phytostabilization and biomonitoring of trace-element contaminated soils
Source: PLoS One. 2017 Jun 30;12(6):e0180240. doi: 10.1371/journal.pone.0180240 (PMC5493371; doi:10.1371/journal.pone.0180240)
Supplement: S4 Table — Range for each element in parenthesis. Abbreviation dl means detection limit. (DOCX) [file pone.0180240.s005.docx]

**S4 Table**. Available concentrations of S and five trace elements at each sampling site (mg kg^-1^; mean values ± SE). Range for each element in parenthesis. Abbreviation dl means detection limit.

| Site | Depth | Cd | Cu | Mn | Pb | S | Zn |
| --- | --- | --- | --- | --- | --- | --- | --- |
| C1 | **0-20** | 0.01 ± 0.02  (0.007-0.015) | 0.13 ± 0.01  (0.11-0.14) | 23.1 ± 2.00  (19.2-25.8) | 0.006 ± 0.005  (0.001-0.015) | 10.7 ± 3.12  (6.07-16.7) | 0.65 ± 0.17  (0.48-0.98) |
|  | **20-40** | 0.01 ± 0.003  (0.005-0.016) | 0.11 ± 0.02  (0.075-0.131) | 16.1 ± 2.38  (12.1-20.4) | 0.06 ± 0.04  (0.001-0.13) | 6.81 ± 2.62  (2.41-11.5) | 0.55 ±0 .12  (0.32-0.70) |
| C2 | **0-20** | 0.002 ± 0.001  (0.001-0.004) | 0.14 ± 0.013  (0.11-0.16) | 0.60 ± 0.33  (0.22-1.26) | 0.08 ± 0.04  (0.001-0.13) | 2.69 ± 1.09  (0.96-4.71) | 0.12 ± 0.006  (0.11-0.13) |
|  | **20-40** | 0.005 ± 0.0001  (0.004-0.005) | 0.13 ± 0.01  (0.11-0.15) | 0.35 ± 0.20  (0.12-0.74) | 0.03 ± 0.03  (0.001-0.1) | 3.47 ± 1.96  (0.25-7.00) | 1.0 ± 0.004  (0.10-0.11) |
| S1 | **0-20** | 0.01 ± 0.002  (0.006-0.012) | 0.16 ± 0.025  (0.12-0.206) | 0.27 ± 0.05  (0.20-0.38) | 0.02 ± 0.013  (0.001-0.04) | 11.4 ± 2.52  (6.90 -15.6) | 0.15 ± 0.043  (0.09-0.23) |
|  | **20-40** | 0.01 ± 0.005  (0.006-0.02) | 0.18 ± 0.02  (0.13-0.21) | 0.30 ± 0.16  (0.09-0.62) | 0.008 ± 0.006  (0.001-0.02) | 16.15 ± 7.57  (1.16-25.5) | 0.28 ± 0.10  (0.14-0.48) |
| S2 | **0-20** | 0.07 ± 0.02  (0.042-0.11) | 0.19 ± 0.02  (0.16-0.23) | 6.81 ± 0.97  (5.40-8.66) | 0.06 ± 0.007  (0.05-0.07) | 46.4 ± 15.8  (28.0-77.7) | 4.19 ± 0.73  (3.03-5.54) |
|  | **20-40** | 0.08 ± 0.03  (0.03-0.14) | 0.60 ± 0.22  (0.17-0.88) | 9.48 ± 5.50  (3.64-20.48) | 0.05 ± 0.03  (0.001-0.08) | 38.0 ± 18.8  (11.5-74.3) | 7.05 ± 0.37  (0.90-13.8) |
| S3 | **0-20** | 0.03 ± 0.003  (0.022-0.033) | 0.25 ± 0.04  (0.21-0.33) | 0.99 ± 0.46  (0.45-1.91) | 0.20 ± 0.19  (0.001-0.59) | 4066 ± 979  (2108-5068) | 0.33 ± 0.04  (0.25-0.40) |
|  | **20-40** | 0.02 ± 0.002  (0.017-0.024) | 0.14 ± 0.02  (0.12-0.17) | 1.99 ± 1.10  (0.62-4.18) | 0.43 ± 0.20  (0.07-0.75) | 5178 ± 83.8  (5070-5342) | 0.31 ± 0.08  (0.15-0.40) |
| S4 | **0-20** | 0.01 ± 0.001  (0.013-0.015) | 0.37 ± 0.05  (0.29-0.47) | 0.41 ± 0.09  (0.29-0.58) | < dl | 448 ± 228  (182-902) | 0.26 ± 0.04  (0.18-0.31) |
|  | **20-40** | 0.02 ± 0.004  (0.018-0.03) | 0.27 ± 0.01  (0.26-0.28) | 0.28 ± 0.09  (0.17-0.45) | 0.023 ± 0.02  (0.001-0.066) | 1668 ± 1330  (94.5-4312) | 0.32 ± 0.13  (0.18-0.56) |
| S5 | **0-20** | 0.02 ± 0.003  (0.013-0.023) | 0.38 ± 0.02  (0.35-0.42) | 0.26 ± 0.01  (0.24-0.29) | 0.02 ± 0.012  (0.01-0.038) | 93.8 ± 47.6  (25.2-185) | 0.30 ± 0.05  (0.22-0.39) |
