# Supplementary material for: Potential of Eucalyptus camaldulensis for phytostabilization and biomonitoring of trace-element contaminated soils
Source: PLoS One. 2017 Jun 30;12(6):e0180240. doi: 10.1371/journal.pone.0180240 (PMC5493371; doi:10.1371/journal.pone.0180240)
Supplement: S5 Table — Range for each element in parenthesis. (DOCX) [file pone.0180240.s006.docx]

**S5 Table**. Macronutrient concentrations in leaves and flower buds at each sampling site (g 100g^-1^; mean values ± SE). Range for each element in parenthesis.

| Organ | site | Ca | K | Mg | N | P | S |
| --- | --- | --- | --- | --- | --- | --- | --- |
| Leaves | C1 | 1.20 ± 0.01  (1.17-1.22) | 0.70 ± 0.02  (0.67-0.75) | 0.29 ± 0.01  (0.28-0.32) | 1.07 ± 0.06  (0.99-1.19) | 0.07 ± 0.01  (0.07-0.09) | 0.13 ± 0.004  (0.12-0.14) |
|  | C2 | 1.67 ± 0.36  (1.25-2.40) | 0.82 ± 0.05  (0.73-0.91) | 0.23 ± 0.03  (0.20-0.29) | 1.38 ± 0.08  (1.26-1.53) | 0.13 ± 0.01  (0.12-0.14) | 0.15 ± 0.006  (0.14-0.16) |
|  | S1 | 2.19 ± 0.58  (1.58-3.35) | 1.07 ± 0.07  (0.97-1.20) | 0.38 ± 0.05  (0.32-0.47) | 1.11 ± 0.05  (1.02 ±1.21) | 0.14 ± 0.02  (0.10-0.18) | 0.14 ± 0.005  (0.13-0.15) |
|  | S2 | 1.10 ± 0.14  (0.83-1.30) | 0.81 ± 0.08  (0.70-0.98) | 0.25 ± 0.006  (0.24-0.26) | 1.08 ± 0.12  (0.85-1.26) | 0.08 ± 0.001  (0.08-0.084) | 0.19 ± 0.02  (0.16-0.23) |
|  | S3 | 1.71 ± 0.07  (1.62-1.85) | 1.29 ± 0.09  (1.14-1.47) | 0.36 ± 0.02  (0.33-0.40) | 1.67 ± 0.13  (1.48-1.92) | 0.16 ± 0.03  (0.11-0.22) | 0.21 ± 0.02  (0.17-0.26) |
|  | S4 | 1.88 ± 0.35  (1.20-2.36) | 1.00 ± 0.08  (0.84-1.13) | 0.31 ± 0.04  (0.26-0.40) | 1.52 ± 0.12  (1.39-1.76) | 0.10 ± 0.004  (0.10-0.11) | 0.17 ± 0.007  (0.16-0.18) |
|  | S5 | 2.58 ± 0.05  (2.48-2.638) | 1.50 ± 0.06  (1.41-1.62) | 0.25 ± 0.02  (0.22-0.30) | 1.63 ± 0.07  (1.52-1.75) | 0.19 ± 0.03  (0.14-0.24) | 0.18 ± 0.01  (0.16-0.21) |
| Flower buds | C1 | 0.85 ± 0.04  (0.78-0.90) | 0.81 ± 0.04  (0.76-0.89) | 0.21 ± 0.02  (0.18-0.23) | 0.61 ± 0.05  (0.52-0.68) | 0.13 ± 0.01  (0.10-0.15) | 0.04 ± 0.003  (0.03-0.04) |
|  | C2 | 0.95 ± 0.09  (0.80-0.10) | 0.82 ± 0.03  (0.75-0.86) | 0.16 ± 0.002  (0.16-0.17) | 0.76 ± 0.10  (0.62-0.96) | 0.15 ± 0.1  (0.13-0.17) | 0.04 ± 0.015  (0.02-0.07) |
|  | S1 | 1.10 ± 0.13  (0.89-1.33) | 0.93 ± 0.03  (0.88-0.99) | 0.16 ± 0.004  (0.16-0.17) | 0.73 ± 0.06  (0.66-0.85) | 0.18 ±0.007  (0.17-0.19) | 0.06 ± 0.017  (0.04-0.10) |
|  | S2 | 0.81 ± 0.08  (0.73-0.97) | 0.88 ± 0.04  (0.80-0.93) | 0.18 ± 0.04  (0.10-0.22) | 0.73 ± 0.04  (0.65-0.78) | 0.15 ± 0.007  (0.14-0.16) | 0.06 ± 0.02  (0.05-0.07) |
|  | S3 | 0.74 ± 0.09  (0.56-0.89) | 1.14 ± 0.10  (0.93-1.26) | 0.17 ± 0.01  (0.15-0.19) | 0.84 ± 0.04  (0.76-0.91) | 0.13 ± 0.006  (0.12-0.14) | 0.07 ± 0.006  (0.06-0.08) |
|  | S4 | 0.71 ± 0.07  (0.56-0.80) | 1.00 ± 0.11  (0.84-1.20) | 0.18 ± 0.01  (0.16-0.20) | 0.85 ± 0.08  (0.72-1.01) | 0.13 ± 0.015  (0.10-0.15) | 0.05 ±0.005  (0.045-0.06) |
|  | S5 | 1.00 ± 0.19  (0.63-1.22) | 1.07 ± 0.07  (0.94-1.18) | 0.14 ± 0.03  (0.12-0.20) | 0.84 ± 0.06  (0.78-0.88) | 0.17 ± 0.01  (0.16-0.19) | 0.06 ± 0.006  (0.05-0.07) |
