# Supplementary material for: Potential of Eucalyptus camaldulensis for phytostabilization and biomonitoring of trace-element contaminated soils
Source: PLoS One. 2017 Jun 30;12(6):e0180240. doi: 10.1371/journal.pone.0180240 (PMC5493371; doi:10.1371/journal.pone.0180240)
Supplement: S6 Table — Range for each element in parenthesis. (DOCX) [file pone.0180240.s007.docx]

**S6 Table.** Trace element concentrations in leaves and flower buds at each sampling site (mg kg^-1^; mean values ± SE). Range for each element in parenthesis.

| Organ | site | As | Cd | Cu | Mn | Pb | Zn |
| --- | --- | --- | --- | --- | --- | --- | --- |
| Leaves | C1 | 0.15 ± 0.14  (0.01-0.43) | 0.05 ± 0.0003  (0.048-0.049) | 10.5 ± 0.23  (10.0-10.9) | 1077 ± 187  (749-1398) | 0.91 ± 0.17  (0.58-1.16) | 28.3 ± 2.85  (24.6-33.9) |
|  | C2 | 0.46 ± 0.14  (0.19-0.62) | 0.005 ± 0.002  (0.002-0.01) | 5.55 ± 0.50  (4.79-6.50) | 75.9 ±14.2  (47.9-94.6) | 0.62 ± 0.11  (0.39-0.73) | 22.0 ± 1.92  (19.2-25.7) |
|  | S1 | 1.49 ± 0.08  (1.33-1.57) | 0.23 ± 0.09  (0.13-0.41) | 16.1 ± 3.22  (9.70-19.6) | 112 ± 9.26  (95.0-127) | 1.64 ± 0.33  (1.14-2.26) | 55.8 ± 11.4  (38.4-77.2) |
|  | S2 | 2.15 ± 0.14  (1.92-2.40) | 0.48 ± 0.05  (0.37-0.54) | 24.7 ± 1.34  (23.2-27.4) | 1500 ± 155  (1235-1771) | 1.47 ± 0.24  (1.08-1.09) | 153 ± 6.27  (144-165) |
|  | S3 | 2.24 ± 0.20  (1.85-2.53) | 0.38 ± 0.10  (0.22-0.56) | 12.6 ± 0.85  (11.6-14.3) | 179 ± 55.8  (82.2-276) | 0.66 ± 0.14  (0.45-0.93) | 90.4 ± 12.2  (69.1-111) |
|  | S4 | 2.96 ± 0.24  (2.52-3.48) | 0.12 ± 0.02  (0.10-0.16) | 10.5 ± 0.86  (9.62-12.2) | 74.5 ± 11.4  (52.7 -91.3) | 0.83 ± 0.04  (0.76-0.90) | 49.5 ± 2.61  (45.7-54.5) |
|  | S5 | 2.19 ± 0.28  (1.62-2.52) | 0.35 ± 0.18  (0.10-0.70) | 11.4 ± 0.98  (9.51-12.8) | 131 ± 36.6  (70.9-196) | 0.93 ± 0.16  (0.63-1.14) | 64.2 ± 15.0  (38.0-90.0) |
| Flower buds | C1 | 0.014 ± 0.003  (0.01-0.02) | 0.07 ± 0.004  (0.06-0.07) | 9.89 ± 1.40  (7.29-12.1) | 645 ± 163  (347-909) | 0.19 ± 0.05  (0.12-0.28) | 17.8 ± 1.61  (15.0-20.5) |
|  | C2 | 0.03 ± 0.01  (0.01-0.05) | 0.04 ± 0.01  (0.01-0.06) | 6.37 ± 0.59  (5.19-6.98) | 52.4 ± 5.43  (44.7-62.9) | 0.18 ± 0.09  (0.005-0.32) | 14.0 ± 0.38  (13.2-14.6) |
|  | S1 | 0.14 ± 0.03  (0.10-0.19) | 0.10 ± 0.02  (0.07-0.14) | 11.4 ± 0.31  (11.0-12.0) | 122 ± 41.8  (70.7-204) | 0.40 ± 0.11  (0.23-0.61) | 31.9 ± 4.79  (26.3-41.5) |
|  | S2 | 0.20 ± 0.02  (0.16-0.23) | 0.44 ± 0.17  (0.15-0.74) | 13.2 ± 1.46  (10.5-15.5) | 692 ± 334  (62.1- 1198) | 0.57 ± 0.07  (0.49-0.70) | 53.6 ± 8.18  (42.0-69.4) |
|  | S3 | 0.29 ± 0.07  (0.19-0.43) | 0.41 ± 0.16  (0.08-0.59) | 12.4 ± 0.95  (11.0-14.3) | 130 ± 42.6  (48.3-194) | 0.37 ± 0.07  (0.23-0.47) | 50.2 ± 9.49  (36.9-68.6) |
|  | S4 | 0.20 ± 0.06  (0.10-0.03) | 0.03 ± 0.01  (0.01-0.06) | 9.72 ± 0.56  (8.98-10.8) | 26.5 ± 0.67  (25.8-27.9) | 0.14 ± 0.12  (0.001-0.38) | 19.6 ± 1.65  (16.7-22.4) |
|  | S5 | 0.26 ± 0.10  (0.06-0.40) | 0.12 ± 0.03  (0.07-0.17) | 11.3 ± 0.89  (9.93-13.0) | 39.6 ± 2.68  (35.5-44.6) | 0.07 ± 0.06  (0.001-0.18) | 25.9 ± 1.44  (24.4-28.8) |
