# Supplementary material for: Potential of Eucalyptus camaldulensis for phytostabilization and biomonitoring of trace-element contaminated soils
Source: PLoS One. 2017 Jun 30;12(6):e0180240. doi: 10.1371/journal.pone.0180240 (PMC5493371; doi:10.1371/journal.pone.0180240)
Supplement: S7 Table — Significant differences per species at each site and organ are marked with and asterisk (p<0.05). (DOCX) [file pone.0180240.s008.docx]

**S7 Table.** Cadmium and Zn concentrations (mg kg^-1^) of *Eucalyptus camaldulensis* and *Salix viminalix* at sites S1 and S2. Significant differences per species at each site and organ are marked with and asterisk (p<0.05)

| Species | Organ | Site | Cd | Zn |
| --- | --- | --- | --- | --- |
| Eucalyptus | Leaves | S1 | 0.24± 0.09* | 55.8 ±11.4 |
|  |  | S2 | 0.48 ± 0.05* | 153 ±6.27* |
| Salix |  | S1 | 3.64 ± 2.10 | 536± 188 |
|  |  | S2 | 7.06 ±2.65 | 935 ± 164 |
| Eucalyptus | Flower buds | S1 | 0.10 ±0.02* | 31.9 ± 4.79* |
|  |  | S2 | 0.44 ±0.17* | 53.6 ±8.19* |
| Salix |  | S1 | 1.28 ± 0.63 | 145 ±28.8 |
|  |  | S2 | 2.48 ± 0.58 | 190 ±14.3 |
